# A β-Hydroxybutyrylation–FOXM1/CENPA Axis Links Ketone-Body Metabolism to Mitotic Transcription in Basal-like Breast Cancer

**DOI:** 10.3390/ijms27157059

**Published:** 2026-08-06

**Authors:** Hugo Tovar, Enrique Hernández-Lemus

**Affiliations:** Computational Genomics Division, National Institute of Genomic Medicine, Mexico City 14610, Mexico

**Keywords:** lysine β-hydroxybutyrylation, master regulator analysis, Basal-like breast cancer, CENPA, FOXM1

## Abstract

Lysine β-hydroxybutyrylation (Kbhb) is a metabolite-derived post-translational modification of histone and non-histone proteins that couples β-hydroxybutyrate (BHB) availability to gene expression. Yet the transcription factors that govern the Kbhb substrate program in cancer remain unidentified. Existing studies have cataloged Kbhb-modified substrates or examined individual proteins, without identifying the transcriptional regulators of the program in a defined tumor context. Here, we performed network-based master regulator analysis (MRA), implemented in the viper package, on a molecular signature restricted to experimentally validated Kbhb substrates, across two independent PAM50 Basal-like breast cancer (BLBC) cohorts profiled on orthogonal platforms: TCGA-BRCA (RNA-seq; n = 195 tumor, 113 normal) and METABRIC (microarray; *n* = 209 tumor, 148 normal). Dataset-specific regulatory networks were inferred with ARACNe-AP and integrated by cross-platform Stouffer meta-analysis. Of 1493 Kbhb substrates, 1322 and 1213 were expressed in the respective cohorts. The analysis identified seven concordant transcriptional master regulators (six activated, one repressed; cross-cohort NES correlation r = 0.64), with CENPA (meta-NES +4.55) and FOXM1 (meta-NES +4.27) as the dominant drivers. These findings nominate a BHB–Kbhb–FOXM1/CENPA axis linking ketone-body metabolism to mitotic transcription, with potentially protumoral implications for ketogenic regimens in BLBC.

## 1. Introduction

Breast cancer remains the most frequently diagnosed malignancy among women, and within it, the triple-negative subtype (TNBC), defined by the absence of estrogen receptor, progesterone receptor, and HER2 expression, carries the poorest prognosis and the fewest targeted treatment options [1]. TNBC, which largely corresponds to the PAM50 Basal-like molecular class, is characterized by a high propensity for early metastasis and a profoundly rewired transcriptional and metabolic state, with dysregulated lipid metabolism increasingly recognized as a hallmark of the subtype [2]. Because the aggressive phenotype of Basal-like tumors is sustained by coordinated, large-scale transcriptional reprogramming rather than by a single driver lesion, the central biological question is not merely which genes are deregulated but which transcription factors orchestrate their deregulation. Identifying the master regulators that govern these programs is therefore a prerequisite for understanding—and ultimately interrupting—the transcriptional logic of the Basal-like phenotype.

One metabolic input now known to act directly on the transcriptional machinery is the ketone body β-hydroxybutyrate (BHB). Produced endogenously during fasting, prolonged exercise, and ketogenic dietary regimens, BHB was long regarded primarily as an energy carrier delivered to peripheral organs, yet it is now appreciated to be far more than a metabolite: it functions as an endogenous inhibitor of histone deacetylases, as a ligand for cell-surface receptors, and as a substrate that links cellular energy state to gene expression [3,4]. A defining example of this signaling capacity is lysine β-hydroxybutyrylation (Kbhb), a post-translational modification in which a β-hydroxybutyryl group is covalently attached to lysine residues [5]. On histones, Kbhb behaves as an activating mark—installed by the acetyltransferase p300 and removed by HDAC1/2—that decorates the promoters of transcriptionally active genes [5]. Its reach extends well beyond chromatin: proteome-wide mapping identified 1493 Kbhb substrates, with marked enrichment in the spliceosome, DNA repair, and chromatin-remodeling machinery [6]. A recently described non-canonical route, in which BHB-derived acetoacetate is converted to cytosolic acetyl-CoA, supplies the acyl donor that p300 uses to deposit these marks and supports tumor growth in vivo, closing the biochemical loop between circulating BHB and the Kbhb program [7].

Converging evidence now implicates Kbhb as a pro-tumoral modification across several cancer types. In TNBC, elevated ketogenesis promotes metastasis through Kbhb of calpastatin, which relieves calpain inhibition and drives epithelial-to-mesenchymal transition [2]. In pancreatic cancer, BHB-driven Kbhb of the transcription factor Snail blocks its ubiquitin-mediated degradation and accelerates metastasis, with circulating BHB clinically associated with disease progression [8]; BHB has likewise been reported to sustain colorectal cancer through metabolic plasticity and apoptosis resistance [9]. Together with the emergence of the writer enzymes p300 and CBP as candidate anticancer drug targets [10], these findings have raised concern that ketogenic diets—often proposed as cancer adjuvants yet supported by inconsistent and rarely subtype-stratified evidence—could be counterproductive in certain settings, including TNBC [1]. Despite this momentum, existing studies have characterized Kbhb either by cataloging modified substrates and testing them for differential expression and pathway enrichment, or by dissecting the modification of individual proteins. None has asked which transcription factors govern expression of the Kbhb substrate program as a whole within a defined tumor context—a question that demands network inference and master regulator analysis rather than substrate cataloging or differential-expression enumeration.

Here, we address that gap by performing network-based master regulator analysis (MRA), implemented in the viper package [11], on a molecular signature deliberately restricted to the experimentally validated Kbhb substrates of Huang et al. [6]. Restricting the signature to the substrates of the modification—rather than to the full tumor-versus-normal expression profile—focuses the analysis on the regulators that specifically govern the Kbhb program instead of the generic regulators of the malignant phenotype, an approach inherited and extended from earlier signature-restricted MRA in breast cancer [12]. We applied this strategy to the Basal-like subtype in two independent cohorts profiled on orthogonal platforms—TCGA-BRCA (RNA-seq; *n* = 195 tumor, 113 normal) and METABRIC (microarray; *n* = 209 tumor, 148 normal)—inferring dataset-specific regulatory networks with ARACNe-AP [13] and integrating the cohorts by cross-platform meta-analysis. Of the 1493 substrates, 1322 and 1213 were expressed in the TCGA and METABRIC Basal cohorts, respectively. This analysis identified seven concordant transcriptional master regulators (six activated and one repressed; cross-cohort NES correlation *r* = 0.64 over 75 common regulators), with CENPA (meta-NES +4.55) and FOXM1 (meta-NES +4.27) as the dominant drivers. These results nominate a BHB–Kbhb–FOXM1/CENPA axis that couples ketone-body metabolism to mitotic transcription, and suggest that ketogenic, BHB-raising regimens could paradoxically reinforce tumorigenic transcription in BLBC.

## 2. Results

### 2.1. The Kbhb Proteome Is Enriched for Transcriptional and Cell-Cycle Regulators in Basal BRCA

To define the transcriptional landscape of lysine β-hydroxybutyrylation (Kbhb) in breast cancer, we compiled a gene set of 1493 experimentally validated Kbhb substrates from the proteomic study by Huang et al. [6], which identified 3248 unique Kbhb sites in 1397 human proteins using immunoaffinity enrichment and LC-MS/MS after β-hydroxybutyrate (BHB) treatment of HEK293 cells.

Gene Ontology analysis of this set revealed significant enrichment for proteins involved in DNA replication, chromosome organization, cell-cycle regulation, and transcriptional control—precisely the protein classes that localize to chromatin and are subject to histone mark-mediated regulation. Of the 1493 Kbhb substrates, 1322 were expressed in the TCGA Basal cohort (log_2_(TPM + 1) > 1 in ≥ 20% of Basal tumors) and 1213 in the METABRIC [14] Basal cohort (log_2_ intensity > 6 in ≥ 20% of Basal samples), establishing a robust, platform-specific gene set for downstream analyses.

### 2.2. ARACNe-AP Networks Capture the Basal-like Regulatory Landscape

Dataset-specific transcriptional regulatory networks were inferred from PAM50 Basal-like tumor expression matrices using ARACNe-AP [13,15] with the 1639 human transcription factors (TFs) defined by Lambert et al. [16] as candidate regulators (100 bootstraps, mutual information *p* < 1 × 10^−8^). The TCGA Basal network (*n* = 195 tumor samples) comprised 183,950 regulatory edges; the METABRIC Basal network (*n* = 209 tumor samples) comprised 110,780 edges. The use of two independent datasets—RNA-seq (TCGA) and Illumina HT-12 microarray (METABRIC)—enabled cross-platform validation of all downstream findings.

### 2.3. CENPA and FOXM1 Are the Top Transcriptional Master Regulators of the Kbhb Program in TCGA

Master regulator analysis (MRA) [17,18] was performed using the msVIPER [11] algorithm with a molecular signature restricted to the expressed Kbhb substrates—rather than the full tumor-versus-normal differential expression profile—to identify TFs that preferentially regulate the Kbhb transcriptional program rather than general oncogenic differences [12]. The t-statistic-based signature was computed by comparing Basal tumor samples (*n* = 195) with solid-tissue normal samples (*n* = 113).

In the TCGA discovery cohort, MYBL2 emerged as the top master regulator (NES = +3.08), followed by CENPA (NES = +2.97), FOXM1 (NES = +2.70), CBX2 (NES = +2.51), and E2F1 (NES = +2.47). HMGA1 (NES = +2.38) and CHCHD3 (NES = +2.33) were also significant. No significantly repressed regulators were identified in this cohort. In total, 13 regulators reached FDR < 0.05 (Supplementary Figure S1A). Shadow analysis [18] revealed a convergent regulatory hierarchy: CENPA, FOXM1, and MYBL2 each independently explain part of the inferred activity of FOXK2, while CBX2 activity is partially explained by MYBL2, suggesting that CENPA and MYBL2 co-occupy the hierarchical apex of the Kbhb regulatory landscape in TCGA (Supplementary Figure S1C).

### 2.4. Independent Validation in METABRIC Confirms the Core TMR Set

MRA was repeated in the METABRIC validation cohort using the ComBat-corrected microarray expression matrix and the independently inferred METABRIC Basal network. CENPA (NES = +3.46) and FOXM1 (NES = +3.33) again ranked first and second among activated regulators, while BNC2 (NES = −3.12), VEZF1 (NES = −3.03), KLF9 (NES = −2.88), and MZF1 (NES = −2.76) were the most strongly repressed. In total, 10 regulators reached FDR < 0.05 in METABRIC (Supplementary Figure S1B). Shadow analysis in this cohort identified CENPA as the primary hierarchical regulator, partially explaining the inferred activity of DNMT1, E2F3, E2F7, and MXD4, while FOXM1 partially explains XBP1 activity (Supplementary Figure S1D). The direction of all seven regulators identified in the cross-platform meta-analysis was concordant across cohorts (NES correlation TCGA vs. METABRIC: *r* = 0.64). This cross-platform concordance demonstrates that the identified TMRs reflect a robust biological program rather than platform-specific noise.

### 2.5. Meta-Analysis Identifies Seven Concordant TMRs; CENPA Is the Hierarchical Master

**Figure 1:**
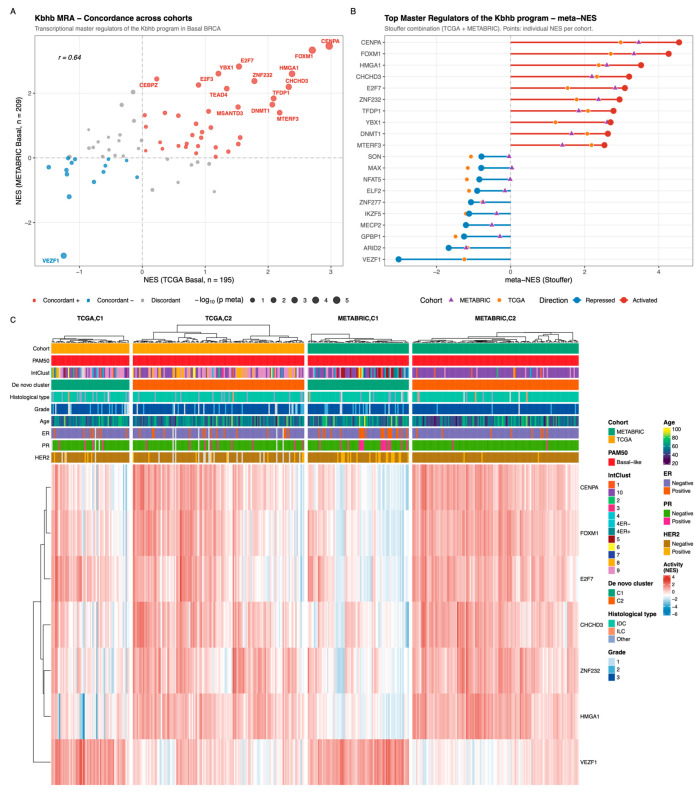
Transcriptional master regulators of the Kbhb program in Basal BRCA. (**A**) Scatter plot of normalized enrichment scores (NES) from msVIPER applied to the Kbhb-restricted molecular signature in TCGA Basal (x-axis, *n* = 195) versus METABRIC Basal (y-axis, *n* = 209). Each point represents one of the 75 regulators detected in both cohorts; color indicates concordance direction (red: concordant activation; blue: concordant repression; gray: discordant); point size encodes −log10(meta *p*-value). Pearson *r* = 0.64. (**B**) Lollipop chart of meta-NES (Stouffer combination) for the top 20 regulators ranked by |meta-NES|. The filled circle represents the meta-NES; overlaid triangles (METABRIC) and circles (TCGA) show the individual cohort NES values. Red segments: activated regulators; blue segments: repressed. The seven regulators reaching FDR < 0.05 are labeled. (**C**) Heatmap of per-sample VIPER activity scores (*z*-scored) for the seven significant Kbhb-axis TMRs (FDR < 0.05), across all Basal-like tumor samples from both cohorts (TCGA, *n* = 195; METABRIC, *n* = 209). Columns are split into four blocks by cohort and de novo activity cluster (C1, C2; Section 4.9) and clustered within each block (1−correlation distance, Ward.D2 linkage); rows (TMRs) are hierarchically clustered. Column annotation tracks (top to bottom): cohort; PAM50 molecular subtype; Integrative Cluster (IntClust, obtained from the original classification for METABRIC and via the iC10 classifier for TCGA); de novo activity cluster; histological type (IDC, ILC, other); histological grade (for METABRIC, from the original clinical annotation; for TCGA-BRCA, derived from the Nottingham component scores of the pathologist-panel re-annotation of Thennavan et al. [33], as grade is not reported in the central GDC clinical data; samples without an assignable grade are shown in gray); age at diagnosis (continuous); and ER, PR, and HER2 status. PAM50 and IntClust track colors are matched to the original publications [14,34]. Generated with ggplot2 [35], ggrepel [36], ComplexHeatmap [37], and patchwork [38].

To integrate MRA results across both cohorts, we applied the Stouffer method to the 75 regulators detected in both datasets, computing a meta-NES as Zmeta = (ZTCGA + ZMETABRIC)/√2, followed by Benjamini–Hochberg multiple-testing correction. Seven regulators reached FDR < 0.05: six activated (CENPA, FOXM1, HMGA1, CHCHD3, E2F7, and ZNF232) and one repressed (VEZF1) (Table 1, Figure 1). CENPA achieved the highest meta-NES (+4.55, FDR = 0.0004), confirming its role as the primary transcriptional master regulator of the Kbhb program in BLBC.

### 2.6. Per-Sample VIPER Activity Reveals Coherent Activation of the Kbhb Program Across Basal Tumors

To assess whether the identified TMRs are consistently active across individual tumors—rather than driven by a subset of samples—we estimated per-sample TF activity using single-sample VIPER, independently in TCGA and METABRIC, with their respective regulons. Activity scores were *z*-normalized per TF within each cohort to account for scale differences between RNA-seq and microarray platforms and then combined into a single TF × sample matrix.

The resulting heatmap (Figure 1C) reveals a stratified activation pattern across the seven significant TMRs. CENPA and FOXM1 show broad, consistent activation across most Basal tumor samples in both cohorts, concordant with their highest meta-NES values. HMGA1 displays a similar but more moderate gradient, with activation evident across most samples but greater inter-tumoral variability. CHCHD3, E2F7, and ZNF232, which carry lower meta-NES values, show a more heterogeneous pattern, as expected from their weaker aggregate signal. VEZF1 presents the inverse profile, with consistently low or negative activity across both cohorts, concordant with its repressed status. Notably, within each cohort, samples were ordered by the mean activity of the activated TMRs, and the resulting left-to-right gradient confirms that this ordering captures a biologically meaningful axis of Kbhb program activity. Taken together, these results indicate that the Kbhb regulatory program is a characteristic—albeit heterogeneous—feature of the Basal-like subtype, with CENPA and FOXM1 as its most robust and consistent drivers.

### 2.7. TMR Activity Segregates Established Molecular Subtypes Within Basal-like Disease

Because PAM50 Basal-like is a heterogeneous category, we annotated the 404 Basal-like samples underlying Figure 1C (195 TCGA, 209 METABRIC) with established subtype classifications: TCGA samples were assigned to the Lehmann/Bareche TNBC molecular subtypes [39,40] (mesenchymal, *n* = 56; immunomodulatory, *n* = 54; basal-like, *n* = 37; mesenchymal stem-like, *n* = 33; luminal AR, *n* = 15), and METABRIC samples were annotated by Integrative Cluster (IntClust) [14], dominated by IntClust 10 (*n* = 143, ~68%), consistent with its established enrichment for Basal-like/Claudin-low disease.

Activity scores of the seven significant Kbhb-axis TMRs (CENPA, FOXM1, HMGA1, CHCHD3, E2F7, ZNF232, VEZF1) were significantly associated with established subtype in both cohorts (ANOVA and Kruskal–Wallis, Benjamini–Hochberg-corrected across 14 tests; all FDR < 0.04, most FDR < 1 × 10^−10^), with the strongest association for CENPA and CHCHD3 (FDR ≈ 1 × 10^−16^ in TCGA; ≈1 × 10^−27^ and 1 × 10^−17^ in METABRIC, respectively). De novo hierarchical clustering of the same seven TMR activity scores (1−correlation distance, Ward.D2 linkage) supported a two-cluster partition (silhouette = 0.493 for k = 2, vs. 0.407 for k = 3 and 0.384 for k = 4), which was highly concordant with the established subtypes (Fisher’s exact test: TCGA FDR = 0.0021, METABRIC FDR < 0.0002). The clusters were likewise concordant with genomic integrative subtype (iC10 for TCGA, IntClust for METABRIC), with C2 enriched for the Basal-associated integrative clusters in both cohorts (Fisher’s exact test: TCGA FDR = 0.0017, METABRIC FDR < 0.001; Supplementary Tables S1, S1b, S2, S2b). The two clusters captured the same activity structure in both cohorts: six of the seven TMRs (all except E2F7) showed concordant differences in mean activity between C1 and C2 in TCGA and METABRIC, with C2 corresponding to high and C1 to low Kbhb-program activity (Supplementary Tables S3).

Because a subset of tumors with a PAM50 Basal-like profile carry hormone receptor or HER2 positivity by immunohistochemistry, we tested whether ER, PR, and HER2 status was associated with de novo cluster membership. In METABRIC, C1 was significantly enriched for receptor positivity relative to C2 across all three markers (ER: 27% vs. 6%; PR: 20% vs. 2%; HER2: 24% vs. 2%; Fisher’s exact test, Benjamini–Hochberg FDR < 0.001 for each). In TCGA, receptor-positivity rates were low and did not differ between clusters (ER: 12% vs. 12%; PR: 7% vs. 7%; HER2: 9% vs. 8%; all FDR = 1). Full contingency counts and test statistics are provided in the Supplementary Tables S4 and S4b, and percentages by cluster and cohort are shown in the Supplementary Figure S2. This cross-cohort discordance indicates that C1 captures a receptor-positive subset of Basal-like tumors specifically within METABRIC, whereas the TCGA Basal-like cohort remains uniformly receptor-low across both de novo clusters. Histological grade tracked the same transcriptional gradient in both cohorts: composite TMR activity increased with grade in METABRIC and, independently, in TCGA (see the composite-score analysis below), with mean composite scores across grades 1–3 of −1.71, −0.66 and 0.10 in METABRIC and −0.29, −0.40 and 0.06 in TCGA; grade 1 comprised only 2 and 4 tumors, respectively, so cohort means at that level are unstable. When the same variable was instead tested against the discrete de novo clusters, the association was recovered in METABRIC, where C1 contained a higher proportion of grade 1–2 tumors than C2 (19.5% vs. 3.1%; Fisher’s exact test, Benjamini–Hochberg FDR < 0.001), but not in TCGA (19.6% vs. 13.5%; FDR = 0.45) (Supplementary Table S5)—consistent with the loss of information incurred by dichotomizing a continuous activity gradient in a cohort in which 85% of tumors are grade 3.

A composite TMR activity score (*z*-scored average of the seven NES values, VEZF1 sign-inverted as the sole repressed regulator; validated against PC1 of the same seven TMRs, *r* = 0.998, 70% variance explained) was positively associated with tumor grade in both cohorts (METABRIC, Spearman ρ = 0.276, FDR = 0.00035; TCGA, ρ = 0.218, FDR = 0.0092) and with tumor stage in METABRIC (ρ = 0.214, FDR = 0.0149), and negatively associated with age at diagnosis in both cohorts (TCGA ρ = −0.196, FDR = 0.0118; METABRIC ρ = −0.133, FDR = 0.067, marginal); no association was found with pathologic stage in TCGA (FDR = 0.628). Neither the continuous composite score (Cox proportional hazards for overall survival: TCGA HR = 0.814, 95% CI 0.549–1.208, FDR = 0.613; METABRIC HR = 0.961, 95% CI 0.803–1.149, FDR = 0.660) nor de novo cluster membership (log-rank: TCGA *p* = 0.093; METABRIC *p* = 0.813) reached significance for overall survival, which we report as a negative finding, noting limited power in TCGA (28 events). Kaplan–Meier curves by de novo cluster are shown in the Supplementary Figure S3.

### 2.8. The Kbhb Transcriptional Program Is Differentially Expressed in Basal Tumors Across Both Platforms

To characterize the transcriptional state of Kbhb substrates in Basal BRCA, we performed differential expression (DE) analysis comparing Basal tumors to normal tissue in each cohort independently: DESeq2 [41] on raw STAR counts for TCGA (*n* = 195 tumors, *n* = 113 normal), and limma [42] on ComBat-corrected [43] log_2_ microarray intensities for METABRIC (*n* = 209 tumors, *n* = 148 normal). A uniform significance criterion (adjusted *p* < 0.05 and |log_2_FC| ≥ 0.5) was applied across both platforms to account for the compressed dynamic range of Illumina microarray data relative to RNA-seq.

**Figure 2:**
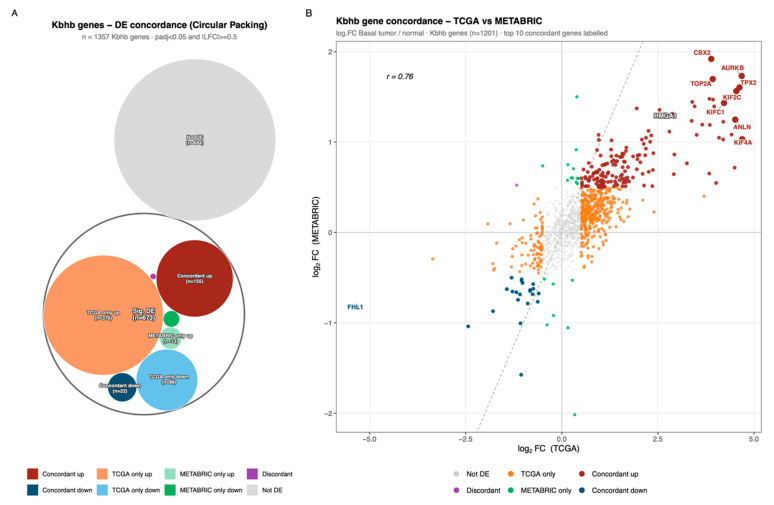
Cross-cohort concordance of Kbhb differential expression in Basal BRCA. (**A**) Circular packing chart of the 1357 Kbhb genes tested for differential expression (adjusted *p* < 0.05 and |log_2_FC| ≥ 0.5). The solid outer circle encompasses all genes with significant DE in at least one cohort (Sig. DE, *n* = 673); the gray circle represents genes without significant DE on either platform (Not DE, *n* = 684). Inner bubbles are area-proportional to gene count and colored by concordance category: concordant upregulated (dark red, *n* = 155), concordant downregulated (dark blue, *n* = 22), TCGA-only up/down (orange/light blue), METABRIC-only (greens), and discordant (purple). Generated with ggraph and tidygraph. (**B**) Scatter plot of log_2_FC values (Basal tumor/normal) in TCGA (x-axis) versus METABRIC (y-axis) for the 1201 Kbhb genes with results in both cohorts. Points are colored by concordance category. The dashed diagonal indicates perfect concordance (slope = 1). Pearson *r* = 0.76. The 10 concordant genes with the highest combined |log_2_FC| are labeled; HMGA1 is highlighted (white text, dark background) as the sole TMR that is simultaneously a concordantly upregulated Kbhb substrate. Generated with ggplot2 and ggrepel.

Of the 1357 Kbhb genes tested in at least one cohort, 673 showed significant DE in at least one platform. Of these, 155 were concordantly upregulated, and 22 concordantly downregulated across TCGA and METABRIC, defining a core Kbhb DE program (Figure 2, Supplementary Figure S4). The larger number of TCGA-only DE genes (475 total: 376 up, 99 down) versus METABRIC-only (≈21) is expected given the greater statistical power of RNA-seq over microarray. The global correlation of log_2_FC values across platforms was *r* = 0.76 (Pearson, *n* = 1201 genes with results in both cohorts), confirming broad cross-platform reproducibility.

Among the seven significant TMRs, all showed evidence of DE in at least one dataset. Notably, CENPA showed the highest statistical significance in TCGA (−log_10_(FDR) ≈ 300), well above FOXM1 (≈200), consistent with its role as the hierarchical master regulator. HMGA1 was the only TMR to qualify as a concordantly upregulated Kbhb substrate—i.e., it is both a regulator and a regulated target of the Kbhb program.

### 2.9. Volcano Plots and Expression Heatmaps Confirm Broad Upregulation of Kbhb Substrates in Tumors

Volcano plots (Supplementary Figure S4A,B) show the distribution of DE results across all Kbhb genes and the superimposed positions of the seven significant TMRs (highlighted in orange). In both cohorts, Kbhb substrates show a net upregulation bias in Basal tumors: in TCGA, 530 Kbhb genes are significantly upregulated versus 122 downregulated; in METABRIC, 168 versus 29. The seven TMRs are visible in the upper region of the volcano plots, confirming their strong and significant differential expression.

Hierarchical clustering of all significantly DE Kbhb genes (TCGA: *n* = 1130; METABRIC: *n* = 913) using (1 − Pearson *r*) distance and Ward D2 linkage (Supplementary Figure S4C,D) reveals a coherent transcriptional pattern: tumor and normal samples segregate into distinct clusters on both platforms, and the dominant Kbhb gene cluster shows elevated expression in tumor, further supporting coordinate upregulation of the Kbhb program in Basal BRCA.

### 2.10. ORA of TMR Regulons Reveals Convergence on Pathways for the Mitotic Cell Cycle and Chromosome Segregation

To characterize the biological functions regulated by each TMR, we performed over-representation analysis (ORA) of their transcriptional regulons (TCGA Basal ARACNe-AP network) against two complementary pathway databases: Gene Ontology Biological Process (GO-BP) and Reactome. GO-BP results were simplified to remove semantic redundancy (similarity cutoff = 0.6). A shared universe of all protein-coding genes in the TCGA Basal expression matrix served as the background.

**Figure 3:**
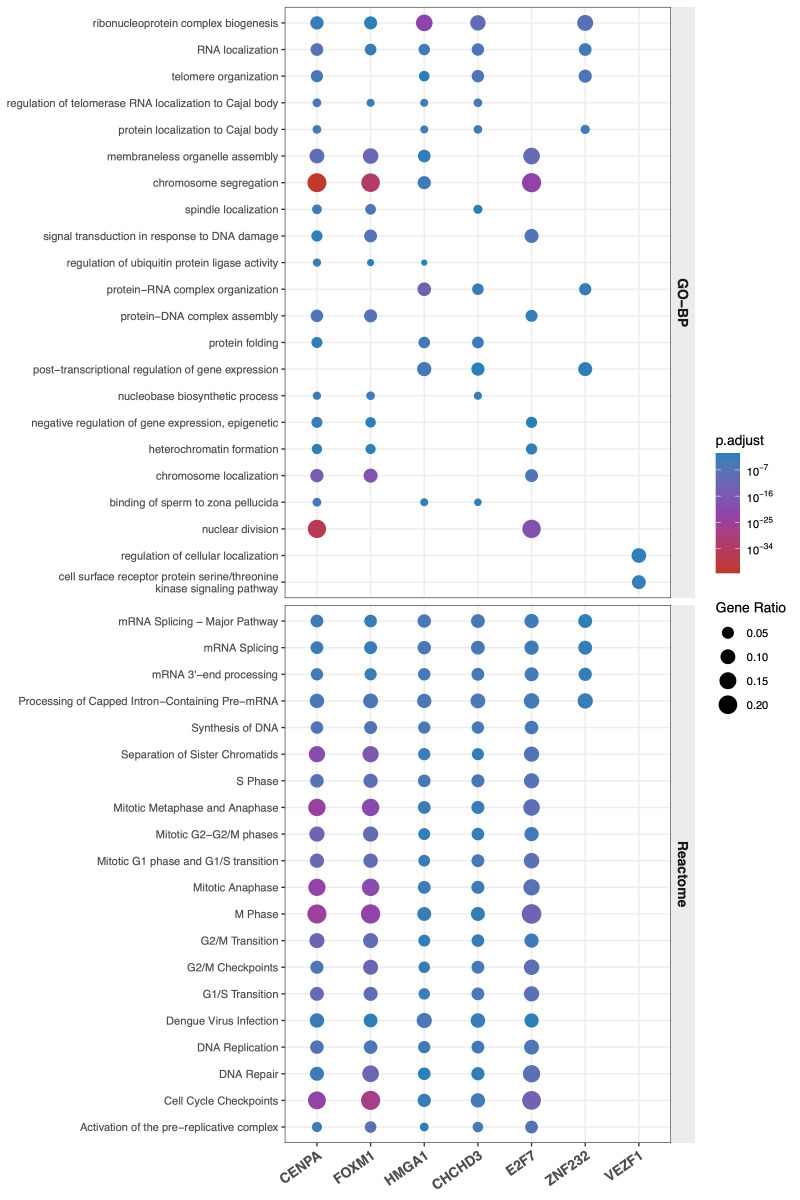
Over-representation analysis (ORA) of the seven significant TMR regulons. Dotplot showing the top 20 pathways most broadly shared across TMR regulons in GO-BP (upper panel) and Reactome (lower panel). The x-axis lists the seven TMRs, ordered left to right by descending meta-NES. Each dot represents a significant enrichment (adjusted *p* < 0.05, *q*-value < 0.2) for that TMR–pathway combination. Dot size encodes the Gene Ratio (fraction of regulon genes annotated to the pathway); dot color encodes the adjusted *p*-value on a log_10_ scale (red: most significant; blue: least significant). GO-BP results were simplified to remove semantic redundancy (similarity cutoff = 0.6). VEZF1, the sole repressed TMR, shows no significant Reactome enrichment and appears without data points in the lower panel. Complete ORA results are provided in the Supplementary Tables S6 and S7. Generated with ggplot2.

The ORA dotplot (Figure 3) shows a striking convergence of the six activated TMR regulons on mitotic cell-cycle pathways. In Reactome, the most widely shared pathways across TMRs include M Phase, Mitotic Metaphase and Anaphase, Separation of Sister Chromatids, G2/M Transition, and Cell Cycle Checkpoints—with CENPA and FOXM1 showing the most significant enrichment (lowest adjusted *p*-values, deepest red in Figure 3). In GO-BP, chromosome segregation, nuclear division, DNA replication, and kinetochore assembly dominate the shared landscape.

Notably, the regulons of CENPA, FOXM1, HMGA1, and CHCHD3 are also enriched for RNA processing terms (mRNA splicing, RNA localization, ribonucleoprotein complex biogenesis), indicating a secondary layer of post-transcriptional regulation within the Kbhb program. VEZF1, the sole repressed TMR, is enriched exclusively in two GO-BP terms (positive regulation of gluconeogenesis; cell surface receptor protein serine/threonine kinase signaling), with no significant Reactome pathway enrichment at FDR < 0.05, consistent with its restricted role in endothelial/vascular identity.

### 2.11. The TMR Regulons Preferentially Target Concordantly Upregulated Kbhb Genes

To integrate network topology with differential expressions, we visualized the ARACNe-AP-supported regulatory edges linking the seven TMRs to their Kbhb DE target genes. All edges present in the TCGA Basal network between a TMR and a Kbhb-DE gene were retained, yielding 436 interactions among 251 unique Kbhb DE target genes.

**Figure 4:**
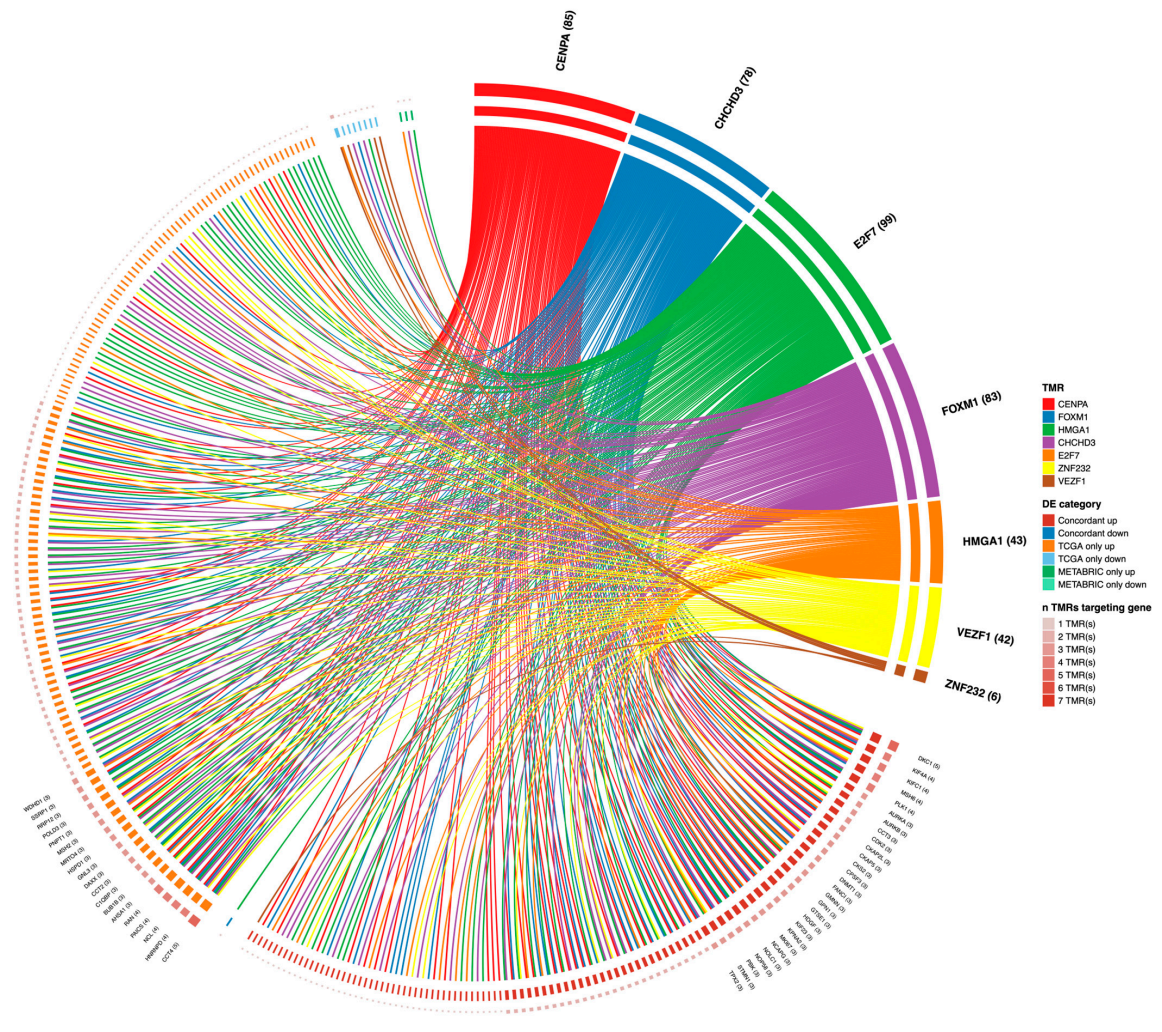
Regulatory interactions between the seven TMRs and differentially expressed Kbhb genes in Basal BRCA (ARACNe-AP TCGA Basal network). Chord diagram with the left arc (upper semicircle) encoding the seven TMRs—sector width proportional to the number of Kbhb DE targets with ARACNe support—and the right arc (lower semicircle) encoding the Kbhb DE genes grouped and colored by concordance category (concordant up: dark red; concordant down: dark blue; TCGA-only up/down: orange/light blue; METABRIC-only: greens). Each chord connects a TMR to a target gene and is colored by the TMR source. The inner track encodes the number of distinct TMRs regulating each gene (gray-to-red gradient). Gene labels (format: SYMBOL (n)) are displayed for genes targeted by ≥3 TMRs. TMR sector labels show the TMR symbol and the total number of Kbhb DE targets. Generated with circlize [44].

The chord diagram (Figure 4) illustrates the architecture of this regulatory network: HMGA1 (99 targets), CENPA (85), and CHCHD3 (83) have the largest regulons among Kbhb DE genes, followed by FOXM1 (78), E2F7 (43), and ZNF232 (42), while VEZF1 has the most restricted overlap (6 targets). Concordantly upregulated genes constitute the dominant category across all six activated TMRs, consistent with coherent transcriptional activation of Kbhb substrates in Basal tumors. A subset of genes is co-regulated by multiple TMRs: genes targeted by three or more TMRs are labeled in the outer track and represent candidate central nodes of the Kbhb regulatory network.

The Sankey diagram (Supplementary Figure S5) quantifies the distribution of ARACNe-supported interactions across TMR and DE categories, confirming that CENPA, FOXM1, E2F7, and CHCHD3 each contribute a substantial proportion of regulatory connections to the concordantly upregulated Kbhb gene set.

### 2.12. The Kbhb-Regulated TMR Panel Shows Graded Specificity Across PAM50 Subtypes

To determine whether the Kbhb→FOXM1/CENPA transcriptional program is specific to Basal-like disease, we repeated ARACNe-AP network inference and msVIPER MRA on PAM50 Luminal A (TCGA *n* = 568, METABRIC *n* = 700), Luminal B (TCGA *n* = 209, METABRIC *n* = 475), and HER2-enriched (TCGA *n* = 82, METABRIC *n* = 224) tumors against the same normal reference groups used for Basal-like, with independently inferred networks per subtype and cohort. Regulon robustness, measured as the number of regulators shared between TCGA and METABRIC, was 75 for Basal-like, 141 for Luminal A, 97 for Luminal B, and 29 for HER2-enriched; the smaller common regulon for HER2-enriched reflects its smaller sample size and warrants cautious interpretation.

**Figure 5:**
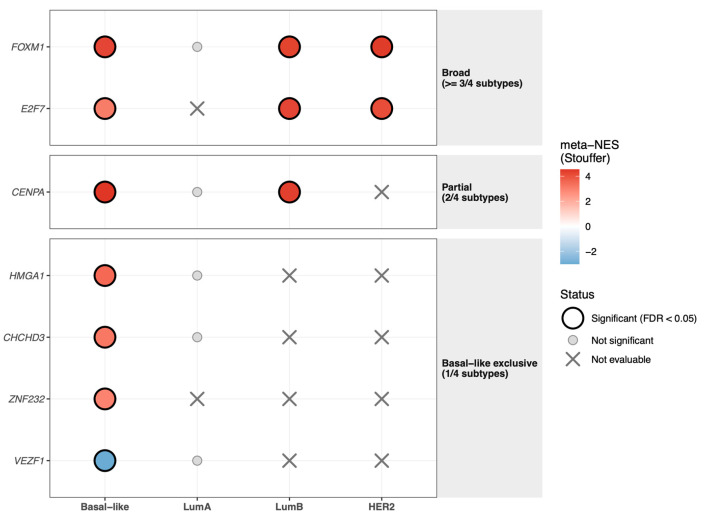
The Kbhb-regulated transcriptional master regulator (TMR) panel shows graded specificity across PAM50 molecular subtypes. Dot plot of meta-NES (Stouffer combination of TCGA and METABRIC) for each of the seven significant Kbhb-axis TMRs across four PAM50 subtypes: Basal-like (reference; TCGA *n* = 195, METABRIC *n* = 209), Luminal A (TCGA *n* = 568, METABRIC *n* = 700), Luminal B (TCGA *n* = 209, METABRIC *n* = 475), and HER2-enriched (TCGA *n* = 82, METABRIC *n* = 224). ARACNe-AP networks and msVIPER MRA were inferred independently for each subtype-by-cohort combination, using the same normal reference group as Basal-like. Dot color encodes meta-NES (red: activated; blue: repressed); dot size/outline encodes significance status: large bordered circles indicate FDR < 0.05 (significant), small pale circles indicate regulators evaluated but not reaching significance, and gray crosses (×) indicate regulators that did not pass the minimum regulon size filter (minsize = 25) or were not among the regulators shared between TCGA and METABRIC in that subtype (not evaluable). TMRs are grouped into three tiers by breadth of significance: broadly active (FOXM1, E2F7; significant in ≥3 of 4 subtypes), partially specific (CENPA; significant in 2 of 4), and Basal-like-exclusive (HMGA1, CHCHD3, ZNF232, VEZF1; significant only in Basal-like). This graded pattern indicates that while FOXM1 and CENPA anchor the hierarchical apex of the Kbhb program within Basal-like disease (Figure 1), specificity for Basal-like breast cancer is most clearly carried by the remaining four regulators of the panel.

FOXM1 retained master-regulator status (meta-FDR < 0.05) in Basal-like, Luminal B, and HER2-enriched, losing significance only in Luminal A (NES = 1.87, FDR = 0.60), consistent with its established role as a pan-cancer proliferation regulator. CENPA was significant in Basal-like and Luminal B, lost significance in Luminal A (NES = 2.11, FDR = 0.49), and did not pass the regulon-size filter in HER2-enriched. The remaining four TMRs (HMGA1, CHCHD3, ZNF232, VEZF1) were significant exclusively in Basal-like, reaching significance in no other subtype. Luminal A showed zero of seven panel TMRs to be significant, indicating the Kbhb-regulated axis is largely inactive in this lowest-proliferation, best-prognosis PAM50 subtype. These results indicate that specificity for Basal-like disease is best understood at the level of the seven-TMR panel rather than for FOXM1 or CENPA individually, since FOXM1 in particular retains broader activity across proliferative subtypes. Full comparative meta-NES values are provided in the Supplementary Table S8 and Figure 5.

## 3. Discussion

The transcriptional consequences of lysine β-hydroxybutyrylation in cancer have so far been examined almost exclusively by cataloging differentially expressed Kbhb substrates and testing them for pathway enrichment, an approach that identifies which genes carry the mark but leaves unanswered the question of who controls their expression. By applying network-based master regulator analysis to a molecular signature restricted to experimentally validated Kbhb substrates [12], this study reframes that question and nominates candidate transcriptional controllers upstream of the Kbhb program rather than its downstream members. The central result is reproducible across two orthogonal platforms: CENPA and FOXM1 are the dominant transcriptional master regulators of the Kbhb program in BLBC, recovered as the two highest-scoring activated regulators in both the TCGA RNA-seq discovery cohort and the METABRIC microarray validation cohort.

CENPA emerged not only as the strongest regulator (meta-NES = +4.55) but also as the hierarchical apex of the inferred network. As a centromeric histone H3 variant, CENPA defines centromeric chromatin epigenetically and is essential for kinetochore assembly and faithful chromosome segregation; its expression peaks at G2/M and is transcriptionally controlled in part by FOXM1 [19]. CENPA is overexpressed in most human cancers, and its levels correlate with tumor aggressiveness, invasiveness, and metastasis in breast cancer [19], while its forced overexpression is causally linked to aneuploidy and genomic instability [20]. Consistent with an organizing role, shadow analysis in METABRIC placed CENPA above DNMT1, E2F3, E2F7, and MXD4, indicating that it partially accounts for the inferred activity of several other regulators. This hierarchical position must, however, be read in light of CENPA’s atypical classification as a transcription factor: it is cataloged as a low-specificity DNA-binding protein on the basis of the centromeric nucleosome structure rather than a sequence-specific motif [16]. Its ARACNe-derived regulon therefore most plausibly reflects co-regulation through chromatin architecture rather than canonical, motif-driven transcription, a distinction that is mechanistically important but does not diminish the coherence of the signal.

**Figure 6:**
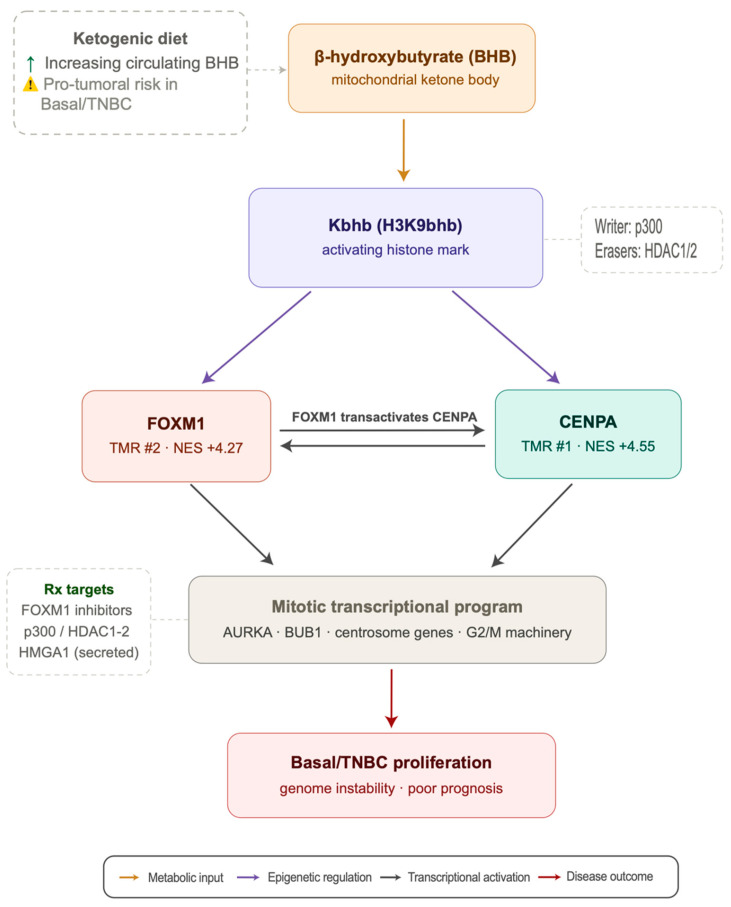
Proposed BHB–Kbhb–FOXM1/CENPA regulatory axis in BLBC. Dashed-outline boxes denote contextual or translational annotations external to the core network—dietary input (upper left), the Kbhb writer/eraser enzymes (right), and candidate pharmacological targets (lower left)—as opposed to the solid-outline nodes and arrows, which depict the BHB–Kbhb–FOXM1/CENPA axis supported by the computational analyses reported here. The green upward arrow indicates increased circulating BHB levels, and the yellow warning symbol denotes a potential pro-tumoral effect. β-Hydroxybutyrate (BHB), produced endogenously or elevated by ketogenic dietary regimens, drives lysine β-hydroxybutyrylation (Kbhb) of histones—a reaction mediated by β-hydroxybutyryl-CoA and catalyzed by the acetyltransferase p300; the mark is removed by HDAC1/2. The resulting H3K9bhb activating mark is enriched at the regulatory targets of FOXM1 and CENPA, the two top transcriptional master regulators (TMRs) of the Kbhb program identified by msVIPER analysis across TCGA and METABRIC (meta-NES: CENPA = +4.55, FOXM1 = +4.27; Stouffer meta-analysis). FOXM1 directly transactivates CENPA, and together they drive a mitotic transcriptional program encompassing AURKA, BUB1, centrosome clustering genes, and broader G2/M machinery, promoting proliferation and genomic instability in BLBC. The elevation of circulating BHB induced by ketogenic dietary regimens may paradoxically reinforce this protumoral axis in BLBC. Pharmacological nodes amenable to therapeutic intervention include FOXM1 (small-molecule inhibitors in preclinical development), the p300/HDAC1-2 writer–eraser pair (existing inhibitor classes), and secreted HMGA1 (antibody-based strategies). NES, normalized enrichment score; TMR, transcriptional master regulator; Kbhb, lysine β-hydroxybutyrylation; BLBC, Basal-like breast cancer.

FOXM1 serves as the canonical counterpart to this chromatin-centric regulator and is proposed to anchor the mechanistic axis described here. It is a master regulator of G2/M progression and the transcription factor most strongly associated with poor survival in breast cancer, particularly in TNBC, while being nearly absent from normal adult tissues [21]. Critically, FOXM1 targets such as CENPA, AURKA, and BUB1 are themselves Kbhb substrates in the Huang proteome [6], and FOXM1 directly transactivates CENPA to drive proliferation, migration, and glycolysis in TNBC cell lines [22], co-upregulating centrosome amplification and clustering genes in this subtype [23]. Because histone Kbhb is reversibly regulated by p300 and HDAC1/2, and H3K9bhb has been linked to active promoter-associated transcription through reader-mediated mechanisms [6,45,46], these observations are consistent with a plausible feedforward model in which elevated BHB may raise Kbhb on the chromatin of FOXM1 and CENPA target genes, potentially reinforcing transcription of the mitotic and centromeric machinery associated with FOXM1 and CENPA activity; this model is inferred from statistical association and has not been directly tested biochemically. The clinical corollary, while indirect, is cautionary. Ketogenic diets, which raise circulating BHB and are being explored as cancer adjuvants, could paradoxically feed this BHB-Kbhb–FOXM1/CENPA axis and act as a protumoral stimulus in BLBC, a concern reinforced by the precedent that BHB accumulation promotes hepatocellular carcinoma through H3K9bhb [46] (Figure 6). This possibility warrants preclinical testing before dietary BHB elevation is considered in this subtype.

The remaining regulators enrich rather than complicate this picture. HMGA1 is unique in being simultaneously an activated TMR and a concordantly upregulated Kbhb substrate: a chromatin architectural protein that drives stem-like, mesenchymal, and metastatic programs in TNBC [24,25]. Its dual status suggests a self-reinforcing node, in which Kbhb modification of HMGA1 and HMGA1-driven transcription of additional Kbhb substrates sustain the program. CHCHD3 is the most unexpected finding: a MICOS-complex inner-membrane protein governing cristae architecture and metabolic homeostasis [27,28], whose appearance as a regulator is best read as a candidate mitochondria-to-nucleus retrograde link consistent with the mitochondrial origin of BHB, although its TF status rests on a single promoter-binding study [47] and demands validation. VEZF1, the sole repressed TMR, is a vascular endothelial factor whose loss aligns with the dedifferentiated phenotype of Basal tumors; given its genome-wide role in modulating Pol II elongation and splicing [32], its repression may relax elongation constraints on Kbhb-regulated RNA-processing genes, a notion compatible with the secondary enrichment of mRNA splicing and ribonucleoprotein biogenesis terms across the CENPA, FOXM1, HMGA1, and CHCHD3 regulons. E2F7 and ZNF232 are best framed as hypotheses: the apparent activation of the atypical repressor E2F7 likely reflects context-dependent co-regulation in the TP53- and RB-altered Basal background and is partially shadowed by CENPA [29,30], whereas the poorly characterized zinc-finger ZNF232 constitutes a genuinely novel prediction requiring experimental follow-up [31].

An important corollary of these results concerns the heterogeneity of the Basal-like intrinsic subtype itself. Consistent with its known imperfect overlap with immunohistochemically defined TNBC [48] (Section 4.2), a minority of PAM50 Basal-like tumors in both cohorts carried ER, PR, or HER2 positivity. Composite TMR activity segregated established molecular subtypes within this population (Lehmann/Bareche TNBC subtypes in TCGA, IntClust in METABRIC; Section 2.7), and receptor positivity itself was unevenly distributed between the two de novo activity clusters—markedly so in METABRIC, where cluster C1 was enriched for ER-, PR-, and HER2-positive tumors relative to C2 (all FDR < 0.001), but not in TCGA, where receptor-positivity rates were low and indistinguishable between clusters. Histological grade, by contrast, tracked the transcriptional gradient consistently in both cohorts, with composite TMR activity increasing with grade in METABRIC and TCGA alike, indicating that the Kbhb-regulated program is coupled to histological aggressiveness irrespective of platform even where the receptor-defined subset is not reproduced. This cross-cohort discordance may reflect differences in cohort composition, IHC scoring practices, or the smaller number of receptor-positive Basal-like tumors available in TCGA, and should be interpreted with appropriate caution rather than as evidence that the Kbhb-TMR axis itself differs by receptor status. It nonetheless reinforces that PAM50 Basal-like is not synonymous with triple-negative disease, and that studies relying on either classification alone risk conflating molecularly distinct tumor subsets.

Several limitations temper these conclusions. The Kbhb gene set derives from HEK293 cells treated with exogenous BHB [6], and tissue-specific substrates in breast epithelium may differ. ARACNe-AP infers co-expression rather than direct binding, so the regulons, especially those of CENPA and CHCHD3, whose DNA-binding evidence is atypical [16], await ChIP-seq or CUT&RUN confirmation. The analysis is observational, leaving the BHB-Kbhb-TMR-phenotype chain to be established by experimental perturbation. More broadly, network-inference-based master regulator analysis identifies transcription factors whose target gene expression patterns are statistically consistent with coordinated regulatory activity; it does not, by itself, establish direct causal or biochemical relationships. These findings should therefore be interpreted as a rigorously supported, cross-cohort computational hypothesis rather than as proof that FOXM1 and CENPA are directly modified by Kbhb or that this modification causally drives mitotic transcription. Direct biochemical demonstration of this modification and its functional consequence remains an important next step. The compressed dynamic range of the METABRIC microarray likely underestimates the Kbhb program. Within these bounds, the genes co-regulated by three or more TMRs represent the highest-confidence core of the network and the most compelling targets for the functional and single-cell studies that should follow.

## 4. Materials and Methods

**Figure 7:**
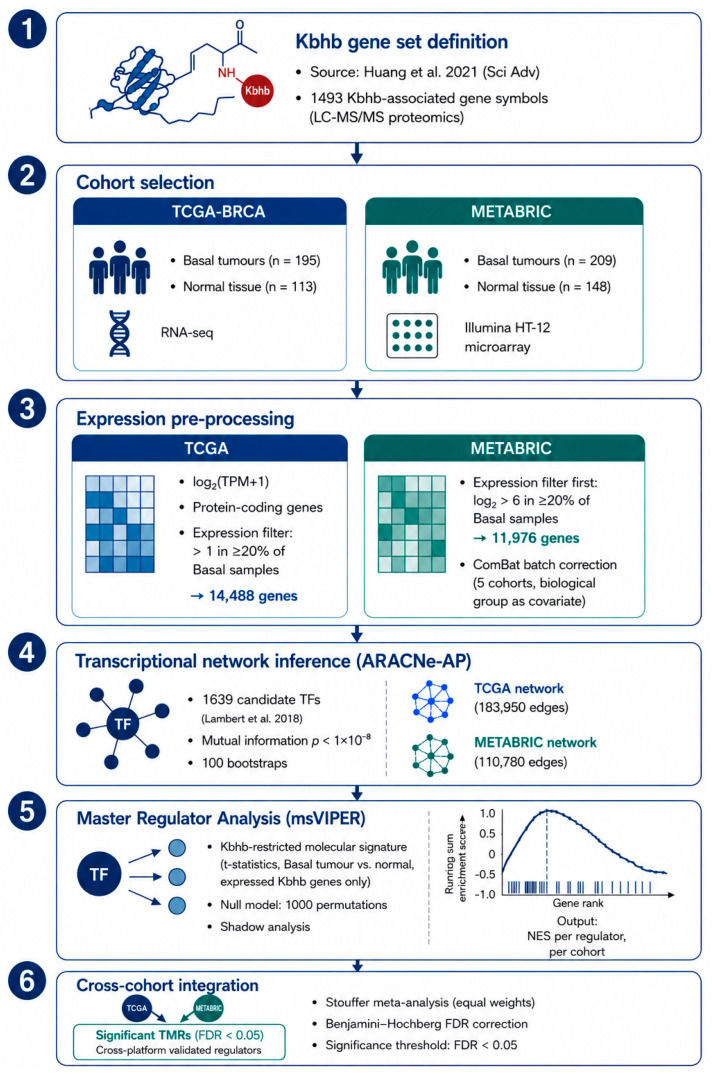
Analytical workflow for identifying transcriptional master regulators of the Kbhb program in BLBC. (1) A gene set of 1493 Kbhb substrates was derived from the proteomic study of Huang et al. [6]. (2) Two independent cohorts were selected: TCGA-BRCA (Basal tumors *n* = 195, normal tissue *n* = 113; RNA-seq) and METABRIC (Basal tumors *n* = 209, normal tissue *n* = 148; Illumina HT-12 microarray). (3) Expression matrices were pre-processed independently per platform: TCGA data were log_2_(TPM+1) transformed and filtered to protein-coding genes expressed in ≥20% of Basal samples (14,488 genes); METABRIC data were first filtered (log_2_ intensity > 6 in ≥20% of Basal samples; 11,976 genes) and then batch-corrected using ComBat (5 cohorts, biological group as covariate). (4) Dataset-specific transcriptional regulatory networks were inferred using ARACNe-AP with 1639 candidate transcription factors [16], mutual information *p* < 1 × 10^−8^, and 100 bootstrap iterations, yielding 183,950 edges (TCGA) and 110,780 edges (METABRIC). (5) Master regulator analysis was performed using msVIPER with a Kbhb-restricted molecular signature (two-sample t-statistics comparing Basal tumor vs. normal tissue, restricted to expressed Kbhb genes), a null model of 1000 permutations, and shadow analysis to resolve regulatory hierarchy. (6) NES values from both cohorts were integrated by Stouffer meta-analysis with Benjamini–Hochberg FDR correction; regulators with FDR < 0.05 were defined as significant cross-platform validated TMRs. Analytical workflow figures were drafted with assistance from OpenAI ChatGPT (GPT-5.5), subsequently reviewed, adapted, and validated by the authors.

The overall analytical workflow is summarized in Figure 7. Briefly, a gene set of experimentally validated Kbhb substrates was derived from a published proteomic dataset [6] and served as the basis for transcriptional master regulator analysis in two independent cohorts of BLBC: TCGA-BRCA (RNA-seq) and METABRIC (Illumina HT-12 microarray). Dataset-specific transcriptional regulatory networks were inferred with ARACNe-AP, and master regulator analysis was performed with msVIPER using a Kbhb-restricted molecular signature. Results from both cohorts were integrated via Stouffer meta-analysis with Benjamini–Hochberg correction to identify transcription factors that consistently regulate the Kbhb transcriptional program across platforms.

### 4.1. Kbhb Gene Set

The Kbhb gene set was derived from the proteomic study by Huang et al. [6], which identified 3248 unique Kbhb sites in 1397 human proteins using immunoaffinity enrichment and liquid chromatography–tandem mass spectrometry (LC-MS/MS) in HEK293 cells treated with 10 mM β-hydroxybutyrate (BHB) for 24 h. Sites were filtered to a false discovery rate (FDR) < 1%, MaxQuant score ≥ 40, and phosphoRS localization probability ≥ 0.75. After mapping to HGNC gene symbols and removing ambiguous entries, the final gene set comprised 1493 unique gene symbols (hereafter referred to as the Kbhb proteome).

### 4.2. Patient Cohorts and Gene Expression Data

TCGA-BRCA (discovery). RNA-seq STAR Counts data for 1231 breast cancer samples were downloaded from the Genomic Data Commons (GDC) portal using the Bioconductor package TCGAbiolinks [49]. Raw counts and transcripts per million (TPM) estimates were retrieved as a SummarizedExperiment object. Clinical annotations, including PAM50 molecular subtype, were obtained from the TCGA Pan-Cancer Clinical Data Resource. The analysis was restricted to PAM50 Basal-like primary tumors (*n* = 195) and solid tissue normal samples (*n* = 113). Only protein-coding genes were retained, and duplicate Ensembl IDs mapping to the same HGNC symbol were resolved by taking the column-wise median.

METABRIC (validation). The METABRIC dataset was downloaded from cBioPortal [50] (brca_metabric; *n* = 2509). Illumina HT-12 microarray expression data (log_2_-transformed) and clinical annotations were obtained. Samples were filtered to those annotated as Basal in the CLAUDIN_SUBTYPE field (*n* = 209) and those annotated as Normal (*n* = 148). Genes with multiple probes were collapsed using the column-wise median.

Sample selection for the Basal-like cohort was based on PAM50 molecular subtyping (TCGA: *n* = 195 Basal-like primary tumors; METABRIC: CLAUDIN_SUBTYPE = “Basal”, *n* = 209) rather than immunohistochemical receptor status. Accordingly, we refer to this cohort as “Basal-like breast cancer (BLBC)” throughout, rather than “triple-negative breast cancer (TNBC),” which is defined by ER/PR/HER2 immunohistochemistry and does not fully overlap with the PAM50 Basal-like intrinsic subtype.

### 4.3. Data Pre-Processing and Normalization

TCGA RNA-seq data were normalized using TPM and a log_2_(TPM + 1) transformation. No additional batch correction was applied because all samples were processed through the GDC harmonized pipeline. Genes with log_2_(TPM + 1) > 1 in at least 20% of Basal tumor samples were retained, yielding 14,488 protein-coding genes.

METABRIC microarray data were already provided on a log_2_ scale. Basal and Normal samples were first filtered for expression (log_2_ intensity > 6 in at least 20% of Basal samples), yielding 11,976 genes. The filtered matrix was then batch-corrected using ComBat from the Bioconductor sva package [43], with cohort identity (five contributing centers) as the batch variable and tumor/normal group membership as a covariate in the model matrix. Correction was applied jointly to Basal and Normal samples before network inference. Tumor/normal group membership was included as a covariate in the ComBat design matrix [51] specifically to protect this biological signal of interest from being removed during batch correction.

### 4.4. Transcriptional Network Inference

Dataset-specific transcriptional regulatory networks were inferred using ARACNe-AP, applied separately to the TCGA Basal (*n* = 195) and METABRIC Basal (*n* = 209) expression matrices. The human transcription factor list from Lambert et al. (*n* = 1639 TFs) was used to define candidate regulators. Network inference was performed with a mutual information *p*-value threshold of 1 × 10^−8^ (DPI tolerance = 0), 100 bootstrap iterations, and a fixed random seed. Bootstrap networks were consolidated using ARACNe-AP’s built-in consolidation step. The final TCGA network comprised 183,950 edges; the METABRIC network comprised 110,780 edges. The mutual information *p*-value threshold and bootstrap design follow standard ARACNe-AP usage [13], and candidate regulators were restricted to the Lambert et al. curated human transcription factor catalog [16]. Bootstrap networks were consolidated using the tool’s default Bonferroni-corrected Poisson test on edge recurrence (*p* = 0.05). To extend this analysis to other PAM50 subtypes (Section 2.12), networks were inferred independently for each of the eight subtype-by-cohort combinations (Basal-like, Luminal A, Luminal B, HER2-enriched × TCGA, METABRIC), avoiding conflation of subtype-specific regulatory structure.

### 4.5. Transcriptional Master Regulator Analysis

Master regulator analysis (MRA) was performed using the msVIPER algorithm implemented in the Bioconductor viper package [11]. For each dataset, the ARACNe-AP network was converted into a regulon object using the *aracne2regulon* function.

The molecular signature was derived by computing two-sample t-statistics for each Kbhb gene expressed in the respective dataset (TCGA: 1322 genes; METABRIC: 1213 genes), comparing Basal tumor samples with matched normal tissue using the rowTtest function. This signature was restricted to Kbhb substrates—rather than the full differential expression profile—to identify transcription factors that preferentially regulate the Kbhb transcriptional program rather than general tumor-versus-normal differences [12]. A null model was generated from 1000 random gene permutations (sampling with replacement, *ttestNull*, seed = 1). Regulators with fewer than 25 targets in the signature were excluded. This null model and minimum regulon size follow standard msVIPER practice [11]. Shadow analysis [18] was performed to identify regulators whose activity is explained by another regulator (regulators = 0.01, shadow = 0.01, targets = 10, per = 1000; package default parameters).

### 4.6. Meta-Analysis Across Datasets

To integrate MRA results from TCGA and METABRIC, regulators detected in both datasets (*n* = 75 in common) were combined using the Stouffer method [52,53]. Because NES values from msVIPER approximate standard normal distributions under the null hypothesis, the meta-NES was computed using the Stouffer method [52,53] as:

$$Z_{\mathrm{meta}}=\frac{Z_{\mathrm{TCGA}}+Z_{\mathrm{METABRIC}}}{\sqrt{2}}$$

Meta *p*-values were derived from the standard normal distribution (*p* = 2·Φ(−|*Z*_meta_|)) and were adjusted for multiple comparisons using the Benjamini–Hochberg procedure. Regulators with FDR < 0.05 were considered significant. This Benjamini–Hochberg threshold was applied uniformly across all statistical layers of the analysis pipeline, consistent with its use for differential expression (Section 4.8) and over-representation (Section 4.11) analyses in this study, providing a single multiple-testing framework throughout.

### 4.7. Per-Sample TF Activity and Visualization

Per-sample transcription factor activity was estimated using the single-sample VIPER algorithm, applied independently to the TCGA and METABRIC expression matrices with their respective regulons (minimum regulon size = 25). Because TCGA (RNA-seq) and METABRIC (microarray) operate on different scales, activity scores were z-score normalized per TF across samples within each cohort before combining. The normalized matrices were then concatenated into a single TF × sample activity matrix for joint visualization. An annotated heatmap was constructed with columns grouped into the four cohort × de novo cluster blocks defined in Section 4.9. Annotation tracks comprised cohort; PAM50 molecular subtype; IntClust, obtained for METABRIC from the original classification [14] and for TCGA via the iC10 classifier [54]; de novo activity cluster (Section 4.9); histological type; histological grade; age at diagnosis; and ER, PR, and HER2 status.

### 4.8. Differential Gene Expression Analysis

#### 4.8.1. TCGA-BRCA

Raw STAR unstranded counts were extracted from the SummarizedExperiment object. Duplicate HGNC symbols were resolved by computing the column-wise median across all rows mapping to the same symbol and rounding to the nearest integer (DESeq2 requirement), consistent with the TPM-based deduplication in Section 4.3. Samples were restricted to PAM50 Basal-like primary tumors (*n* = 195) and solid tissue normal samples (*n* = 113). Genes with ≥10 counts in at least 20% of samples were retained before modeling. Differential expression was estimated using DESeq2 with the design formula ~condition (Tumor vs. Normal). Wald test *p*-values were adjusted for multiple comparisons using the Benjamini–Hochberg procedure (α = 0.05).

#### 4.8.2. METABRIC

Differential expression was performed directly on the ComBat-corrected log_2_ microarray intensities (Section 4.3) using the limma package. A design matrix was constructed with *model.matrix(~condition)*. Linear models were fitted with lmFit, and moderated *t*-statistics were computed with eBayes. Adjusted *p*-values were obtained via topTable using the Benjamini–Hochberg method.

### 4.9. Basal-like Subtype, De Novo Clustering, and Clinical Association Analysis

Established molecular subtypes were assigned per cohort: TCGA Basal-like samples were classified into the five stable Lehmann/Bareche TNBC subtypes [39,40], and METABRIC IntClust assignment was taken from the cBioPortal clinical annotation [14,50]. Association with Kbhb-TMR activity (NES) was tested per TMR and cohort (ANOVA and Kruskal–Wallis, 14 tests, BH-corrected). A composite activity score (mean *z*-scored NES across the seven significant TMRs, VEZF1 sign-inverted, validated against PC1) was correlated with clinico-pathological variables (Spearman’s rank correlation) and tested against overall survival (univariate Cox regression), both BH-corrected. De novo activity clusters were defined by hierarchical clustering of the seven TMR scores (1−correlation distance, Ward.D2 linkage), with k chosen by maximizing average silhouette width; concordance with established subtype, receptor status (ER/PR/HER2), histological grade, and genomic subtype (IntClust, including the TCGA iC10 classification of Section 4.7) was tested with Fisher’s exact test (Monte Carlo-simulated *p*-values) and, for IntClust, the chi-squared test as a sensitivity check, all Benjamini–Hochberg-corrected within each family of tests; survival by cluster was assessed with Kaplan–Meier estimates and the log-rank test. Full contingency tables are reported in the Supplementary Tables S1, S1b, S2, S2b, S4, S4b and S5. Histological grade for TCGA-BRCA is not reported in the central GDC clinical annotation and was instead derived from the pathologist-panel re-annotation of Thennavan et al. [33]. Nottingham scores were computed as the sum of the tubule formation, nuclear pleomorphism and mitotic count component scores reported in that study, and converted to grade (3–5, grade 1; 6–7, grade 2; 8–9, grade 3). Of the 195 TCGA Basal-like samples, 188 corresponded to patients included in the re-annotated cohort, and 182 had all three components scored; the remaining 13 (7 not re-annotated, 6 with at least one component missing) are shown as missing and were excluded from the corresponding test. Monte Carlo *p*-values were computed with B = 10,000 replicates under a fixed random seed set immediately before each simulation, so that results are reproducible and independent of execution order.

### 4.10. Cross-Cohort Concordance of Kbhb Differential Expression

To assess the reproducibility of the Kbhb transcriptional response across platforms, a consensus table was constructed from all Kbhb genes present in at least one cohort’s DE results (*n* = 1357 genes). Genes were assigned to one of six mutually exclusive categories based on significance (adjusted *p* < 0.05 and |log_2_FC| ≥ 0.5) and direction in each cohort: Concordant up (significant and upregulated in both), Concordant down (significant and downregulated in both), Discordant (significant in both but in opposite directions), TCGA only, METABRIC only, and Not DE. Of the 673 genes with DE evidence in at least one cohort, 155 were concordantly upregulated and 22 concordantly downregulated across both platforms.

### 4.11. Over-Representation Analysis of Significant TMR Regulons

To characterize the biological functions regulated by each of the seven significant TMRs, over-representation analysis (ORA) was performed on their transcriptional regulons using two complementary pathway databases.

#### 4.11.1. Gene Lists

For each significant TMR, the set of target genes was extracted from the TCGA Basal ARACNe-AP regulon object. Target gene symbols were converted to Entrez IDs using the *bitr* function from the clusterProfiler package [55] with the Homo sapiens annotation database (org.Hs.eg.db). Genes without a unique Entrez ID mapping were discarded. The background universe was defined as all protein-coding genes in the TCGA Basal expression matrix with a valid Entrez ID.

#### 4.11.2. GO Biological Process

ORA against the Gene Ontology Biological Process (GO-BP) database was performed using the compareCluster function (clusterProfiler) with fun *= “enrichGO”*, ontology “BP”, Benjamini–Hochberg *p*-value adjustment (pvalueCutoff = 0.05, qvalueCutoff = 0.2), and readable = TRUE. To reduce redundancy introduced by the hierarchical structure of the GO-BP ontology, the resulting term set was simplified using clusterProfiler::simplify (similarity cutoff = 0.6, ranked by *p*.adjust).

#### 4.11.3. Reactome

ORA against the Reactome pathway database was performed using *compareCluster* with fun = “enrichPathway” from the ReactomePA package [56] (organism = “human”, same *p*-value and q-value thresholds, readable = TRUE). No simplification step was applied, as Reactome uses a non-redundant hierarchical structure at the pathway level.

### 4.12. Network Visualization of TMR–Kbhb Gene Interactions

To integrate network topology with differential expression, ARACNe-AP edges in the TCGA Basal network were filtered to retain interactions in which the regulator was one of the seven significant TMRs and the target was a differentially expressed Kbhb gene (adjusted *p* < 0.05 and |log_2_FC| ≥ 0.5 in at least one cohort). This yielded 436 TMR–gene interactions covering 251 unique Kbhb DE target genes.

### 4.13. Software and Reproducibility

Bioinformatic analyses were performed in R across two computing environments. Primary analyses—data acquisition, pre-processing, network inference, msVIPER, Stouffer meta-analysis, and differential expression (DESeq2/limma)—were executed in R version 4.5.2 on a Linux server (2 × Intel Xeon Platinum 8368, 152 cores, 1 TiB RAM). Over-representation analysis, expression heatmaps, and regulatory network diagrams (circos and Sankey) were generated locally in R version 4.6.0. Key package versions—server: TCGAbiolinks 2.38.0 [49], SummarizedExperiment 1.40.0 [57], sva 3.58.0 [43], viper 1.44.0 [11], DESeq2 1.50.2 [41], limma 3.66.0 [42], clusterProfiler 4.18.4 [55], ggplot2 4.0.3 [35], ggrepel 0.9.8 [36], patchwork 1.3.2 [38], ggraph 2.2.2 [58], tidygraph 1.3.1 [59]; local (R 4.6.0): clusterProfiler 4.20.0 [55], ReactomePA 1.56.0 [56], org.Hs.eg.db 3.23.1 [60], pheatmap 1.0.13 [61], ComplexHeatmap 2.26.1 [37], circlize 0.4.18 [44], iC10 2.0.2 [54], iC10TrainingData 2.0.1. ARACNe-AP was run as a standalone Java application on the Linux server. Analysis scripts are available at [https://github.com/hachepunto/kbhb_mra] (accessed on 22 July 2026).

## 5. Conclusions

This work introduces a Kbhb-substrate-restricted master regulator analysis framework that moves beyond cataloging differentially expressed modification sites to identify the transcription factors that control their expression. It reveals CENPA and FOXM1 as the hierarchical apex of the β-hydroxybutyrylation transcriptional program in BLBC across two independent platforms. The coherence of this program, anchored by a feedforward BHB–Kbhb–FOXM1/CENPA axis that couples metabolic state to mitotic and centromeric gene expression, suggests that elevated BHB—as occurs under ketogenic dietary regimens—could paradoxically reinforce tumorigenic transcription in BLBC. Beyond the canonical oncogenes, ZNF232 emerges as a consistently co-activated but functionally uncharacterized regulator, making it a priority candidate for the first experimental dissection of its role in cancer. The pharmacological tractability of FOXM1 [62] and chromatin-modifying enzymes such as p300/CBP and class I HDACs [6,10], together with emerging evidence that ketogenesis or BHB exposure can promote TNBC metastasis and breast-cancer cell survival under treatment [1,2,63], translates these computational findings into concrete hypotheses for therapeutic and dietary-intervention research.

## Figures and Tables

**Table 1:** Significant transcriptional master regulators (TMRs) of the Kbhb program in Basal BRCA.

Regulator	NES TCGA	NES METABRIC	Meta-NES	FDR	Biological Relevance
**CENPA**	+2.97	+3.46	+4.55	0.0004	Centromeric histone H3 variant. Overexpressed in cancer and linked to genome instability, kinetochore function and cell-cycle progression; consistent with overlap between the Kbhb program and chromosomal machinery [19,20]
**FOXM1**	+2.70	+3.33	+4.27	0.0007	Master regulator of G2/M progression. Well-documented oncogene in breast cancer/TNBC; FOXM1 has been linked experimentally to CENPA-dependent TNBC proliferation, migration and glycolysis, and to centrosome/cell-cycle programs [21,22,23]
**HMGA1**	+2.38	+2.60	+3.52	0.011	Chromatin architectural protein. Promotes oncogene transcription and tumor progression in TNBC, with roles in stem-like phenotypes, migration/invasion and cell-cycle/histone-gene regulation. Sole TMR that is also a concordantly upregulated Kbhb substrate [24,25,26]
**CHCHD3**	+2.33	+2.19	+3.20	0.026	Inner mitochondrial membrane protein (MIC19/CHCHD3). Core MICOS-associated factor required for crista integrity and mitochondrial function; supports the hypothesis that this TMR links BHB-driven metabolic state with mitochondria-to-nucleus signaling [27,28]
**E2F7**	(no sig.)	+2.82	+3.08	0.031	Atypical E2F repressor of G1/S transcription. Reported to repress oscillating cell-cycle genes and control S-phase progression; its apparent activation here may reflect context-dependent co-regulation of DNA-replication/cell-cycle targets shared with CENPA. Subject to shadow correction by CENPA in METABRIC [29,30]
**ZNF232**	+1.78	+2.38	+2.94	0.035	Poorly characterized C2H2 zinc-finger/SCAN-domain protein predicted to participate in transcriptional regulation; original structural and expression analysis supports nuclear localization and broad tissue expression. Novel prediction requiring experimental validation [31]
**VEZF1**	−1.25	−3.03	−3.02	0.031	Vascular endothelial zinc-finger factor. REPRESSED—consistent with loss of endothelial/vascular identity in Basal BRCA; VEZF1 is linked to endothelial development/angiogenesis and genome-wide transcriptional regulation [32]

NES: normalized enrichment score (msVIPER). meta-NES: Stouffer-combined score. FDR: Benjamini–Hochberg-adjusted meta *p*-value. Positive NES indicates activation; negative indicates repression.

## Data Availability

The TCGA-BRCA dataset is publicly available via the Genomic Data Commons (https://portal.gdc.cancer.gov/) (accessed on 22 July 2026). The METABRIC dataset is available through cBioPortal (https://www.cbioportal.org/) (accessed on 22 July 2026). Analysis code, ARACNe-AP regulatory networks, and master regulator analysis result tables are publicly available in the GitHub repository (https://github.com/hachepunto/kbhb_mra, release v1.3-minor-revision-2) (accessed on 28 July 2026) and archived at Zenodo: https://doi.org/10.5281/zenodo.20768838 (all versions).

## Supplementary Materials

The following supporting information can be downloaded at: https://www.mdpi.com/article/10.3390/ijms27157059/s1.
