# Supplementary material for: A β-Hydroxybutyrylation–FOXM1/CENPA Axis Links Ketone-Body Metabolism to Mitotic Transcription in Basal-like Breast Cancer
Source: Int J Mol Sci. 2026 Aug 6;27(15):7059. doi: 10.3390/ijms27157059 (PMC13467039; doi:10.3390/ijms27157059)
Supplement: Supplementary file 1 [file ijms-27-07059-s001.zip › ijms-4418547_supplementaryFigures.pdf]

Supplementary figures

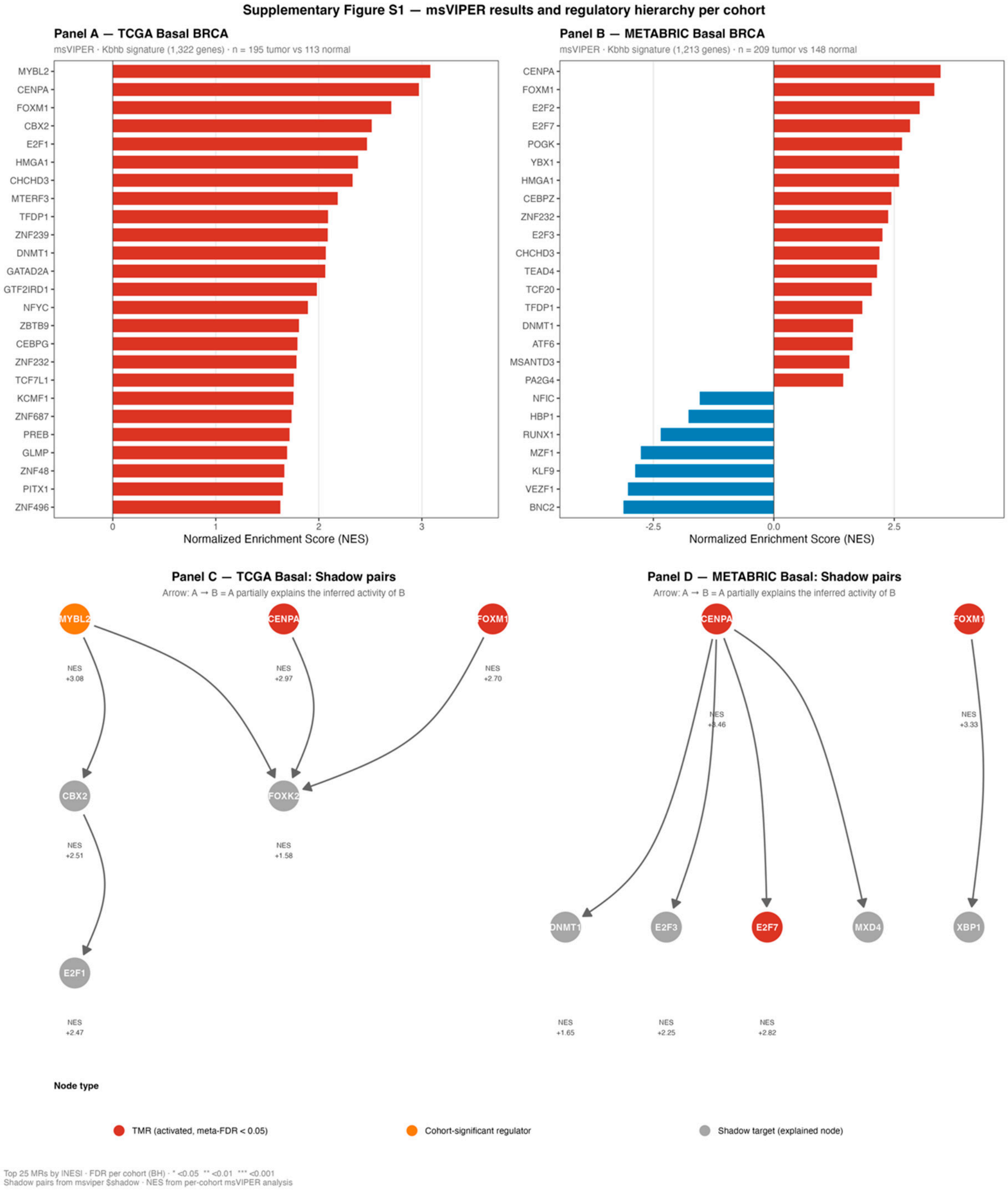

Figure S1. msVIPER master regulator analysis results and regulatory hierarchy per cohort. (A) Top 25 transcriptional master regulators (TMRs) of the Kbhb transcriptional program in TCGA Basal BRCA (n = 195 tumor vs. 113 normal; Kbhb signature: 1,322 genes), ranked by Normalized Enrichment Score (NES). (B) Top 25 TMRs in METABRIC Basal BRCA (n = 209 tumor vs. 148 normal; Kbhb signature: 1,213 genes). In panels A and B, NES was estimated by msVIPER using

cohort-specific ARACNe-AP regulons and a molecular signature restricted to expressed Kbhb substrates. Red bars indicate activated regulators (NES > 0); blue bars indicate repressed regulators (NES < 0). Significance thresholds after Benjamini–Hochberg correction: * FDR < 0.05, ** FDR < 0.01, *** FDR < 0.001. **(C)** Shadow analysis regulatory hierarchy in TCGA. **(D)** Shadow analysis regulatory hierarchy in METABRIC. In panels C and D, arrows indicate shadow relationships (A → B: regulator A partially explains the inferred activity of B). Node color indicates classification: red = cross-platform significant TMR (meta-FDR < 0.05); orange = cohort-significant regulator; grey = shadow target. NES values shown below each node correspond to the per-cohort msVIPER analysis. Shadow network generated with ggraph [Pedersen 10.32614/cran.package.ggraph] and tidygraph [Pedersen tidygraph].

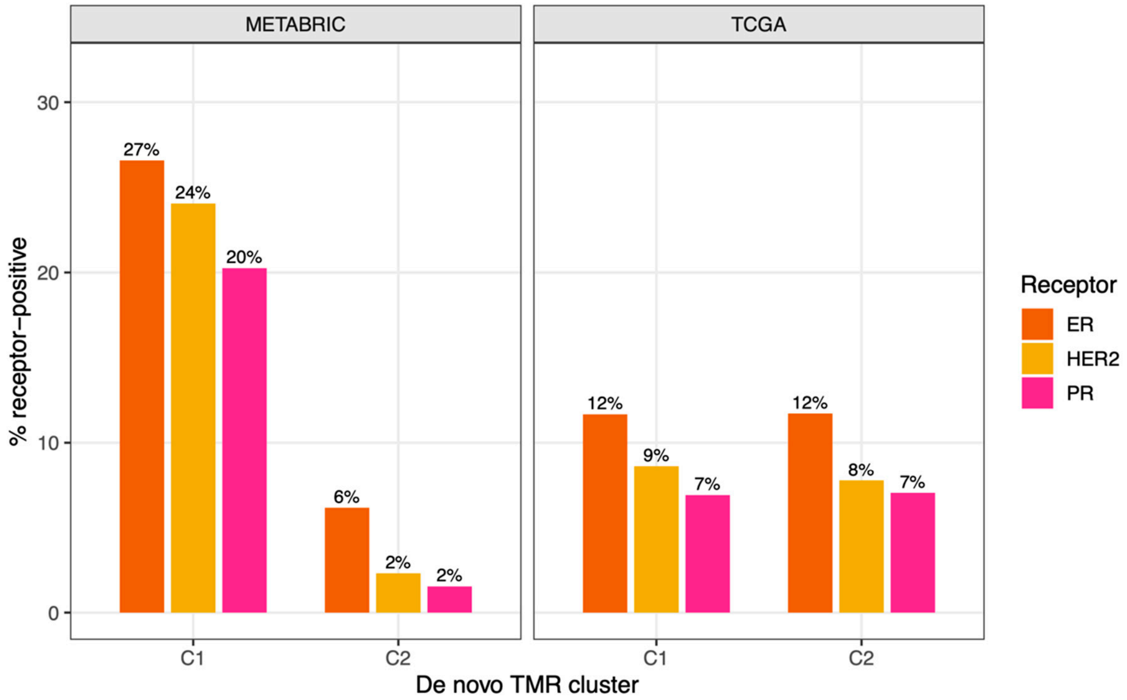

Figure S2. Receptor-positivity rates by de novo activity cluster within Basal-like breast cancer. Bar plots show the percentage of ER-, PR-, and HER2-positive tumors within each de novo cluster (C1, C2; as defined in Figure S3) for METABRIC (left) and TCGA-BRCA (right), with percentages labeled above each bar. Sample sizes per receptor reflect tumors with available annotation (TCGA: ER n = 188, PR n = 186, HER2 n = 174; METABRIC: n = 209 for all three receptors). Association between cluster and receptor status was tested with Fisher's exact test (simulated p-value) and Benjamini–Hochberg FDR correction across the six tests (Supplementary Table S4; contingency counts in Supplementary Table S4b). C1 was significantly enriched for receptor positivity in METABRIC (ER, PR, HER2: all FDR < 0.001) but not in TCGA (all FDR = 1). Generated with ggplot2.

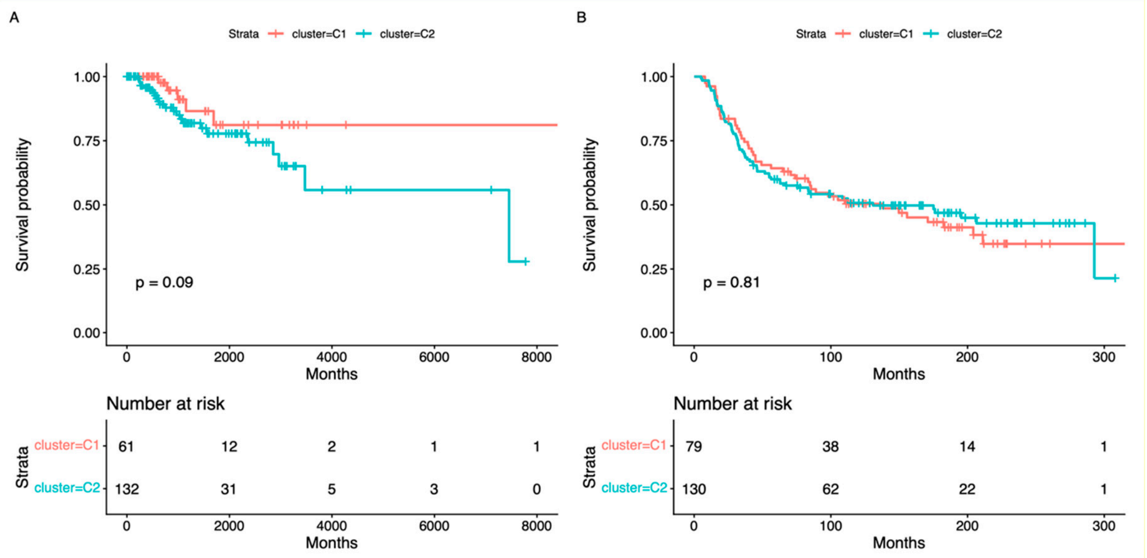

Figure S3. Kaplan-Meier overall survival curves by de novo activity cluster within Basal-like breast cancer. Samples were stratified into two clusters (C1, C2) by hierarchical clustering of the seven significant Kbhb-regulated TMR activity scores (1−correlation distance, Ward.D2 linkage; silhouette width = 0.493 for k=2). (A) TCGA-BRCA Basal-like tumors (n=193; C1 n=61, C2 n=132; log-rank p=0.093). (B) METABRIC Basal-like tumors (n=209; C1 n=79, C2 n=130; log-rank p=0.813). Neither cohort showed a significant association between de novo cluster and overall survival.

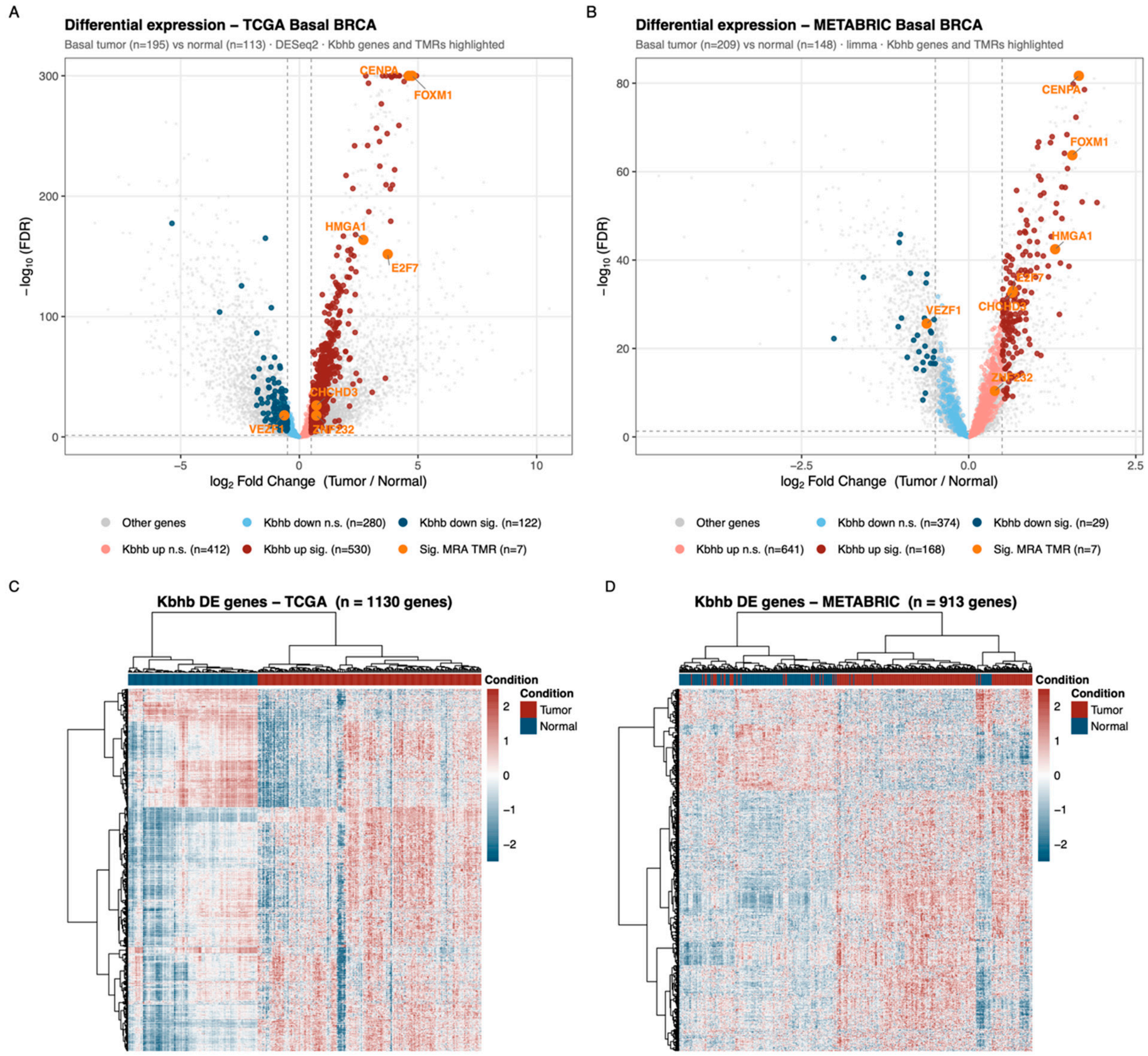

Figure S4. Differential expression of Kbhb genes in Basal BRCA. (A–B) Volcano plots of differential expression (Basal tumor vs. normal tissue) in TCGA-BRCA (A, DESeq2; n = 195 tumor, 113 normal) and METABRIC (B, limma; n = 209 tumor, 148 normal). Kbhb genes are colored by significance and direction: significantly upregulated (dark red), significantly downregulated (dark blue), non-significant (light shades). The seven significant TMRs from the meta-analysis are shown in orange and labeled. Dashed lines indicate the adjusted p = 0.05 and |log2FC| = 0.5 thresholds. Generated with ggplot2 and ggrepel. (C–D) Heatmaps of z-score-normalized log2 expression for all significantly DE Kbhb genes in TCGA (C, n = 1,130 genes) and METABRIC (D, n = 913 genes). Rows: Kbhb DE genes; columns: individual samples annotated by group (Tumor: dark red; Normal: dark blue). Hierarchical clustering uses (1 − Pearson r) distance and Ward D2 linkage. Generated with pheatmap [Kolde pheatmap].

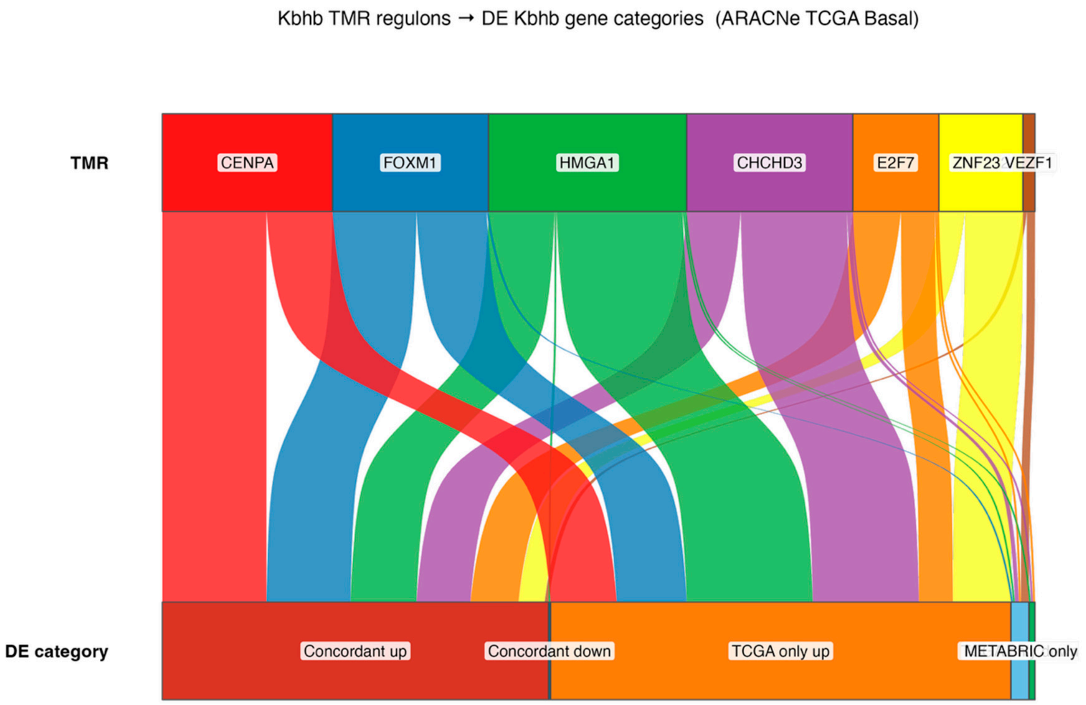

Figure S5. Quantitative distribution of ARACNe-supported TMR–Kbhb gene interactions by DE category. Sankey (alluvial) diagram with the upper axis representing the seven TMRs (ordered by meta-NES, left to right) and the lower axis representing the six DE concordance categories of Kbhb target genes. Flow width is proportional to the number of ARACNe edges between a TMR and a DE category. TMR colors match Figure 4; DE category colors match Figure 2. Generated with ggalluvial [Brunson 10.21105/joss.02017].
